# Supplementary material for: Provider anticipation and experience of patient reaction when deprescribing guideline discordant inhaled corticosteroids
Source: PLoS One. 2020 Sep 17;15(9):e0238511. doi: 10.1371/journal.pone.0238511 (PMC7498097; doi:10.1371/journal.pone.0238511)
Supplement: S1 File — (DOCX) [file pone.0238511.s001.docx]

S1 File. Unexposed provider interview guide.

*Grounded prompts:* If responses are limited or require clarification, probes may be used to elicit more detailed responses. Probes should use words or phrases presented by the participant using one of the following formats:

*What do you mean by ____________?*

*Tell me more about ____________?*

*Can you give me an example of ____________?*

*Tell me about a time when ____________?*

*Can you tell me who ____________?*

*Can you clarify the type of inhaler __________?*

Throughout the interview we’ll be referring to inhaled corticosteroids as ICS. Is this okay?

- What is your current position?

*[If needed]* What are your main responsibilities?

*[If needed]* When did you start this role?

- How do you define mild COPD?

[*If needed*] How do you define moderate COPD?

- Tell me about your experience prescribing ICS for mild COPD.
- Tell me about your experience prescribing ICS for moderate COPD.
- Have you had patients who have ICS prescriptions from other providers?

*[If Yes]* Tell me about discontinuing ICS with these patients.

[If needed] Have you had patients who request ICS prescriptions based on recommendations from non-VA providers?

*[If needed]* When another provider has prescribed ICS, how do you decide whether to discontinue a prescription?

- Tell me about the evidence related to prescribing ICS for mild COPD.

*[If needed]* Tell me about the evidence related to prescribing ICS for moderate COPD.

- How clear are the guidelines for prescribing ICS for COPD?

This study is looking at improving patient safety through De-Implementing ICS for Mild COPD by recommending that providers discontinue prescribing ICS for mild-to-moderate COPD.

- Are you familiar with this recommendation?
- What is your impression of this recommendation?
- Tell me about the evidence related to discontinuing the prescribing of ICS for mild-to-moderate COPD.
- Who should be involved in reducing unnecessary prescribing of ICS in patients with mild-to-moderate COPD?

*[If needed]* How should they be involved?

- Please describe the patient’s role in discontinuing ICS.

*[If needed]* What attitudes or preferences do patients express regarding ICS for mild-to-moderate COPD?

- How do you think your patients with mild-to-moderate COPD would respond to discontinuing ICS?

*[If needed]* Can you give me an example of a time when you discontinued ICS with a patient, and the discussion you had with the patient?

- Do you have any questions for us, or is there anything else you would like to add?
