# Supplementary material for: Provider anticipation and experience of patient reaction when deprescribing guideline discordant inhaled corticosteroids
Source: PLoS One. 2020 Sep 17;15(9):e0238511. doi: 10.1371/journal.pone.0238511 (PMC7498097; doi:10.1371/journal.pone.0238511)
Supplement: S2 File — (DOCX) [file pone.0238511.s002.docx]

S2 File. Intervention-exposed provider interview guide.

*Grounded prompts:* If responses are limited or require clarification, probes may be used to elicit more detailed responses. Probes should use words or phrases presented by the participant using one of the following formats:

*What do you mean by ____________?*

*Tell me more about ____________?*

*Can you give me an example of ____________?*

*Tell me about a time when ____________?*

*Can you tell me who ____________?*

*Can you clarify the type of inhaler __________?*

We will be referring to the program as DISCUSS.

- What is your current position?

*[If needed]* What are your main responsibilities?

What is your role with discontinuing ICS?

When did you start this role?

- Please tell me about your experience prescribing ICS for mild to moderate COPD.
- Please tell me about your experience with the DISCUSS program.

How satisfied have you been with the DISCUSS program?

Have you received recommendations regarding inhalers from other providers?

*[As needed]* How well does DISCUSS fit with your practice?

- How aware do you think other prescribers at your site are regarding the recommendations to stop using ICS?

Who else at your site do you discuss reducing the use of ICS with?

How receptive do you think other providers at your site have been to reducing the use of ICS?

- *[As needed]* How easy or difficult has it been to follow through recommendations to stop prescribing ICS for mild to moderate COPD?

Were there any challenges to following through on recommendations to stop using ICS?

What, if anything, made following through on recommendations to stop using ICS easier?

- Was there anything that surprised you about stopping the use of ICS?
- Have you had patients who have ICS prescriptions from other providers?

*[If yes]* Tell me about discontinuing ICS with these patients.

Have you had patients who request ICS prescriptions based on recommendations from non-VA providers?

When another provider has prescribed ICS, how do you decide whether to discontinue a prescription?

- Tell me about the effect of discontinuing ICS on patient care.

Tell me about the patient experience of de-implementing ICSs.

- Please describe the evidence related the use of ICS for mild to moderate COPD?

What are the risks associated with ICSs?

What alternatives to ICSs are available?

*[If needed]* Please describe the evidence related to not using ICS for mild to moderate COPD?

- How useful was the training for de-implementing ICS?
- What types of unintended consequences have you noticed from discontinuing ICS?
- Please describe the patient’s role in determining ICS use?
- What attitudes or preferences do patients express regarding ICS use?
- How do you think your patients with mild to moderate COPD would respond to discontinuing ICS?

Can you give me an example of a time when you discontinued ICS with a patient, and the discussion you had with the patient?

- How have you felt about receiving these orders from Pulmonology? [Feasibility & Sustainability]
- Would you like to see pulmonologists continue to provide this kind of proactive outreach in the form of unsigned orders?
- At your facility, how involved has leadership been in the DISCUSS program?
- Is there anything else you would like us to know about these practices changes? Is there anyone else you would suggest we talk to regarding this project?
- Do you have any questions for us?
