# Supplementary material for: Provider anticipation and experience of patient reaction when deprescribing guideline discordant inhaled corticosteroids
Source: PLoS One. 2020 Sep 17;15(9):e0238511. doi: 10.1371/journal.pone.0238511 (PMC7498097; doi:10.1371/journal.pone.0238511)
Supplement: S3 File — (DOCX) [file pone.0238511.s003.docx]

S3 File. Intervention-exposed patient interview guide.

*Grounded prompts:*  If responses are limited or require clarification, probes may be used to elicit more detailed responses. Probes should use verbatim words or phrases presented by the participant using one of the following formats:

*What do you mean by ____________?*

*Can you tell me more about ____________?*

*Can you give me an example of ____________?*

*Can you tell me about a time when ____________?*

- Do you see a doctor about your breathing?
- Do you use any inhalers for your breathing?

*[If yes]*

a. What is the type or name of the inhalers you use?

b. Under what circumstance do you use your inhalers(s)?

c. How often do you use your inhaler?

*[If no]*

a. Have you used inhalers in the past?

b. What type or name of the inhalers did you use?

c. Under what circumstance did you use your inhalers(s)?

d. When did you stop using your inhaler?

- Has there been a time you wanted an inhaler and you did not have it?
- Has your inhaler prescription changed within the last few months?

*[If yes]* Tell me about the change.

- Can you tell me about a time when you got too much care?
- Can you tell me about a time when you didn’t get enough care?
- Is there anything we should have asked you about inhalers that we did not?
- Do you have any questions for us, or is there anything else you would like to add?
