# Supplementary material for: Provider anticipation and experience of patient reaction when deprescribing guideline discordant inhaled corticosteroids
Source: PLoS One. 2020 Sep 17;15(9):e0238511. doi: 10.1371/journal.pone.0238511 (PMC7498097; doi:10.1371/journal.pone.0238511)
Supplement: S4 File — (DOCX) [file pone.0238511.s004.docx]

**S4 File. Consolidated criteria for reporting qualitative studies (COREQ): 32-item checklist**

Developed from: Tong A, Sainsbury P, Craig J. Consolidated criteria for reporting qualitative research (COREQ): a 32-item checklist for interviews and focus groups. *International Journal for Quality in Health Care*. 2007. Volume 19, Number 6: pp. 349 – 357

| **No. Item** | **Guide questions/description** | **Authors’ Responses** | **Reported on Page #** |
| --- | --- | --- | --- |
| **Domain 1: Research Team and Reﬂexivity** | | | |
| *Personal Characteristics* | | | |
| 1. Interviewer/facilitator | Which author/s conducted the interview or focus group? | CG, GGS, KCS, and Scott Wanner | Qualitative interviews (Page 8) and Acknowledgements (Page 23) |
| 2. Credentials | What were the researcher’s credentials? E.g. PhD, MD | MD, PhD, PsyD, MA, and BA | Title page (Page 1) |
| 3. Occupation | What was their occupation at the time of the study? | TJP is a VA HSR&D Fellow and a senior fellow at University of Washington Division of Gerontology and Geriatric Medicine; CG is a Health Services Researcher. GGS is a Health Services Researcher and Assistant Clinical Professor (University of Washington, Public Health); KCS is a Research Health Scientist, and Scott Wanner was a volunteer Research Assistant. All non-faculty positions are at in the Veterans Administration’s Health Services Research and Development. | Title page (Page 1) |
| 4. Gender | Was the researcher male or female? | CG, GGS, and SW are male; TJP and KCS are female. | N/A |
| 5. Experience and training | What experience or training did the researcher have? | CG is a qualitative researcher and analyst who holds a PhD is Sociology. GGS holds a PsyD Clinical Family Psychology and is the Director of a VA HSR&D Center of Innovation Qualitative Research Core and has supervised, trained and mentored multiple MD and Students, PhD fellows, career development wards, investigators and staff. KCS has an MA in Psychology with an emphasis in Marriage and Family Therapy and received 4 years of supervision and training in qualitative methods from GGS. SW has undergraduate coursework in qualitative methods and was supervised by GGS as part of a one-year volunteer research assistant position. | N/A |
| *Relationship with participants* | | | |
| 6. Relationship established | Was a relationship established prior to study commencement? | Some participants interviewed were clinician-researchers that have worked with members of the qualitative team (GGS & KCS) in the past on other research projects. The interviewers were not working with participants on any projects at the time that the interviews were completed. No participants were involved in this project in any way. | N/A |
| 7. Participant knowledge of the interviewer | What did the participants know about the researcher? e.g. personal goals, reasons for doing the research | Participants were informed that the researchers were part of a quality improvement project regarding the use of inhalers (veterans and providers) and COPD (providers). | supplemental files:   - S1 File. Unexposed provider interview guide. - S2 File. Intervention-exposed provider interview guide. - S3 File. Intervention-exposed patient interview guide. |
| 8. Interviewer characteristics | What characteristics were reported about the inter viewer/facilitator? e.g. Bias, assumptions, reasons and interests in the research topic | A brief introduction about the interviewers including organizational affiliations, their names and positions were provided to the participants before starting the interview. No interviewer-related biases were identified. | supplemental files: S1 File, S2 File, and S3 File. |
| **Domain 2: Study Design** | | | |
| *Theoretical framework* | | | |
| 9. Methodological orientation and Theory | What methodological orientation was stated to underpin the study? e.g. grounded theory, discourse analysis, ethnography, phenomenology, content analysis | Inductive and deductive content analysis (as described by Elo & Kyngäs) | Qualitative Interviews (Page 9) |
| *Participant selection* | | | |
| 10. Sampling | How were participants selected? e.g. purposive, convenience, consecutive, snowball | Participants were purposively sampled.  Providers who transferred/left the participating VHA facilities or changed positions were excluded. Resident physicians were excluded from the overall project due the needed continuity of contact with the intervention. Patients whose medical record indicated active suicidal or aggressive behaviors, cognitive issues, homelessness, current inpatient hospitalization, or co-occurring interventions (i.e. current enrollment in COPD research) were excluded. | Participants and Recruitment (Page 7-8) |
| 11. Method of approach | How were participants approached? e.g. face-to-face, telephone, mail, email | Providers were contacted by VA email. Veterans were recruited by mail and telephone. | Participants and Recruitment (Page 7-8) |
| 12. Sample size | How many participants were in the study? | We conducted 16 interviews with providers unexposed to the intervention; 6 interviews with intervention-exposed providers; and 9 interviews with patients. | Results (Page 11) |
| 13. Non-participation | How many people refused to participate or dropped out? Reasons? | Unexposed Providers   - 177 invitations were emailed - 20 were excluded - 25 declined - 116 did not respond to invitations   Intervention-exposed providers   - 58 invitations were emailed - 0 were excluded - 8 declined - 44 did not respond to invitations   Intervention-exposed patients   - 82 were screened by chart review - 52 were excluded - 30 invitations were emailed - 6 declined - 15 did not respond to invitations | supplemental file:   - S5 Table. Interview participants |
| *Setting* | | | |
| 14. Setting of data collection | Where was the data collected? e.g. home, clinic, workplace | All interviews were conducted by telephone. Provider interviews were conducted during their work hours. | Participants and Recruitment (Page 8) |
| 15. Presence of non-participants | Was anyone else present besides the participants and researchers? | No | N/A |
| 16. Description of sample | What are the important characteristics of the sample? e.g. demographic data, date | Gender (provider and Vet), Race (Vet) and Provider type (Provider). Providers were all outpatient primary care providers. | Table 1 (Page 12) |
| *Data Collection* | | | |
| 17. Interview guide | Were questions, prompts, guides provided by the authors? Was it pilot tested? | Interview guides with open-ended questions and semi-structured probes. Probes used participants’ words and phrases to elicit details. Interview guides were not pilot-tested but updated iteratively for clarity. | Qualitative Interviews (Page 8-9) and supplemental files: S1 File, S2 File, and S3 File. |
| 18. Repeat interviews | Were repeat inter views carried out? If yes, how many? | No | N/A |
| 19. Audio/visual recording | Did the research use audio or visual recording to collect the data? | Interviews were audio recorded. | Qualitative Interviews (Page 8) |
| 20. Field notes | Were ﬁeld notes made during and/or after the interview or focus group? | No field notes were made. Written interview notes were made to assist the interview process and identify technical or other issues regarding data collection quality. No issues were identified. | N/A |
| 21. Duration | What was the duration of the inter views or focus group? | Interviews ranged from approximately 20-30 minutes. | Qualitative Interviews (Page 8) |
| 22. Data saturation | Was data saturation discussed? | Yes | Qualitative Interviews (Page 8) and Limitations (Page 22) |
| 23. Transcripts returned | Were transcripts returned to participants for comment and/or correction? | No | N/A |
| **Domain 3: Analysis and Findings** | | | |
| *Data Analysis* | | | |
| 24. Number of data coders | How many data coders coded the data? | Three authors (CG, KCS and TJP) coded the data. | Qualitative Interviews (Page 9) |
| 25. Description of the coding tree | Did authors provide a description of the coding tree? | No, authors utilized codes and code groups to identify and generate domains and categories. | N/A |
| 26. Derivation of themes | Were themes identiﬁed in advance or derived from the data? | Barriers and facilitators to de-implementation, Awareness of Evidence, and Use of Substitutions were a-priori categories. | Qualitative Interviews (Page 9) |
| 27. Software | What software, if applicable, was used to manage the data? | AtlasTi | Qualitative Interviews (Page 9) |
| 28. Participant checking | Did participants provide feedback on the ﬁndings? | No | N/A |
| *Reporting* | | | |
| 29. Quotations presented | Were participant quotations presented to illustrate the themes/ﬁndings? Was each quotation identiﬁed? e.g. participant number | Yes, quotations were presented to illustrate the themes/findings, and each quotation was identified with an anonymous participant identifier. | Results (Pages 13-18) and supplemental file:   - S6 Table. Matrix of quotes relevant to patient reaction. |
| 30. Data and ﬁndings consistent | Was there consistency between the data presented and the ﬁndings? | Yes, there was consistency between the data presented and the findings. | Discussion (Pages 19-22) |
| 31. Clarity of major themes | Were major themes clearly presented in the ﬁndings? | Yes, major themes were clearly presented in the Results section using specific sections regarding each theme. | Table 2 and Results (Pages 13-18) |
| 32. Clarity of minor themes | Is there a description of diverse cases or discussion of minor themes? | No | N/A |
