# Supplementary material for: Provider anticipation and experience of patient reaction when deprescribing guideline discordant inhaled corticosteroids
Source: PLoS One. 2020 Sep 17;15(9):e0238511. doi: 10.1371/journal.pone.0238511 (PMC7498097; doi:10.1371/journal.pone.0238511)
Supplement: S1 Table — (DOCX) [file pone.0238511.s005.docx]

| **Respondent Type** | **Total Invitations** | **Excluded** | **Declined** | **Non-response** | **Completed Interviews** |
| --- | --- | --- | --- | --- | --- |
| Unexposed Providers | 177  NP=43  PA=7  Physicians=89  Unknown=38 | 20 | 25 | 116 | 16  NP=2  PA=0  Physicians=14 |
| Intervention-exposed Providers | 58  NP=15  PA=1  Physician=33  Unknown=9 | 0 | 8 | 44 | 6  NP=0  PA=0  physicians=6 |
| Patients | 30* | 0 | 6 | 15 | 9 |

S1 Table: Interview participants

*Prior to interview invitation, 82 intervention-exposed patients were screened by chart review and 52 were excluded based on the criteria discussed in the methods.
