# Supplementary material for: Provider anticipation and experience of patient reaction when deprescribing guideline discordant inhaled corticosteroids
Source: PLoS One. 2020 Sep 17;15(9):e0238511. doi: 10.1371/journal.pone.0238511 (PMC7498097; doi:10.1371/journal.pone.0238511)
Supplement: S2 Table — (DOCX) [file pone.0238511.s006.docx]

S2 Table. Matrix of quotes relevant to patient reaction.

Table S2a: Matrix of quotes anticipating varying patient reaction from providers who were not exposed to the intervention.

|  | **Unexposed Provider Quotes Anticipating Patient Resistance or Reaction** | **Unexposed Provider Quotes Anticipating Acceptance** |
| --- | --- | --- |
| **Pro 101** | -- | -- |
| **Pro 102** | -- | -- |
| **Pro 103** | "My impression is that it’s hard to get somebody off of something if they’re doing well." | "I don’t think people are totally wedded to their inhalers but it’s really hard to get people off of medications that are potentially addictive." |
| **Pro 104** | -- | "For the most part, they’d like to be on the least amount of medications that work and don’t give them side effects." |
| **Pro 105** | -- | -- |
| **Pro 106** | "But, if the patient is really insisting that they’re helpful, I would tend to continue them." | -- |
| **Pro 107** | "ten percent [of patients] do have strong opinions, sometimes logical, sometimes not so logical, on the use of particular medications." | -- |
| **Pro 108** | -- | "...they’re generally, totally on board with it. Especially when it’s easier for them to do." |
|  |  | "I think the majority of patients understand that medical knowledge changes over time and as we learn more we need to use that new information to make our practice better." |
|  |  | "...there’s a combination inhaler, two medications for COPD that is once a day. It’s easier to be compliant with than the old way we were doing things. It certainly makes it a lot easier to make that change." |
| **Pro 109** | "When you’re taking something away, they may think that they need it, so you have to convince them that it’s not actually helping them." | -- |
| **Pro 110** | -- | -- |
| **Pro 111** | "If they say ‘I’ve been on my Advair. I love my Advair. it makes me feel better’ then I would prescribe it, but I generally don’t start with it." | "...if they’re not aware of the downsides of corticosteroids then they’re generally receptive to listening about it, and if I have an opinion about it, like I think we should try you on Albuterol or on an anticholinergic instead, then they’re generally receptive to it." |
|  | "Some have tried other medications and they feel like [ICS] work for them, and they really like their corticosteroid. If that’s the case, as long as they understand the risks and benefits, then I continue." |  |
| **Pro 112** | "Because a lot of times they’ll come in with [a non-VA] prescription for Advair…And we have to struggle, we’re the ones that are supposed to write the prescription part…The patients want it. So a lot of times, you fight and fight with the [non-VA] doctors which takes a lot of time. Or the provider gives in and just writes for it. A lot of time, you don’t have time to fight. It’s a mess…" | -- |
|  | "It’s more of a crutch. I think that’s part of it. And any pharma change, especially with people that have a hard time breathing, they look at it as ‘oh my God, if you change this, I’m going to be short of breath and wind up in the ER’. So it begins." |  |
| **Pro 113** | -- | -- |
| **Pro 114** | -- | "*It’s shared decision-making… patient usually go along with it.* They ask about side effects. They ask if they will get worse… I give them the opportunity to tell me if they do feel worse. There’s not a lot of resistance frankly." |
| **Pro 115** | -- | "I’ve never had any trouble getting people to stop taking them. They taste terrible, people get thrush. I think folks have to be pretty miserable to be enthusiastic about an inhaled steroid." |
| **Pro 116** | -- | -- |

16 total unexposed interviews. Of these, 6 did not bring up reactance or acceptance. Two declined to be recorded (ICS2010, ICS2015). Key: Alternating color coded represent a different speaker to help visualize the range of quotes said by each speaker. Italics represent a potential strategy used to aid in deprescribing ICS. “--” Indicate no applicable quotes were found from the interview.

Table S2b: Matrix of quotes regarding patient reactions from providers were exposed to the intervention

|  |  | **Exposed Provider Quotes Experiencing Resistance/Reactance** | **Exposed Provider Quotes Experiencing Acceptance** |
| --- | --- | --- | --- |
| **Pro 201** |  | "Because they feel like something is working very well so they don’t want to change it, even though they may not have the exact indication for it." | "Some patients have some inhaler loyalty, but once they try the intervention, they seem to be willing. |
|  |  | "They’re just worried that the new inhaler won’t work as well as the old inhaler. That they’ll be short of breath and they’ll be uncomfortable…I think they probably feel it’s working and they ask me, ‘if it’s not broke, why do we need to fix it?’" | "I think everyone I’ve had to take off of ICS has been amenable to it, *but sometimes I can convince them in 5 or 10 minutes, others it might take a month or two, so I keep talking to them."* |
|  |  | "Some of the veterans, they’re pretty stuck on some of their treatments. And it could’ve been something that some great doc told them they needed to do that was 12 years ago, and *it just may take some time to get a new doctor that they trust*." |  |
|  |  | "I guess for patients that are reluctant to switch it’s just, I don’t know why, maybe they’re just so afraid of change they just don’t want something taken away from them. And they feel like they’re doing well enough that they don’t understand sometimes why we need to change." |  |
|  |  | "[I have one patient who] just loves his Symbicort. I guess I’m not going to fix that, I’m stuck with that. Every time he comes in I bring it up with him though, I do." |  |
| **Pro 202** |  | "If I’m not the one prescribing it, a lot of times they’ll want to check with their doctor first or they’ll be hesitant to make a change." | "I think they were all pretty receptive to making a switch. *With the caveat that if it was significantly worse with the switch, then we could always put them back on inhaled corticosteroid*." |
|  |  |  | "If they don’t need the medication, there’s not much point in prescribing something that’s only going to do them harm. I think most of my patients were very receptive to that." |
| **Pro 203** |  | "Some of my patients have wondered if they truly need to stop something that seems to be working for them, and I understand that mindset, but that’s not been particularly surprising either." | *"It’s like with anything, if you have an open dialogue and conversation about it, and aren’t too heavy handed,* I think patients are pretty receptive." |
|  |  | "I think the trickiest part, like I said, at least from what I’ve seen so far, is just for someone that’s been really stable and who hasn’t been hospitalized who’s feeling fine, if it ain’t broke why fix it? That’s sort of the attitude that you have to move around... *There needs to absolutely be buy-in and if there’s not buy-in, then it’s sort of pointless to do any of these medication things.*" |  |
| **Pro 204** |  | -- | "*I tell them that there’s shown to be some increased risk of pneumonia on these medications, so that if we don’t think they absolutely need it, that we should take them off of it…*(patients responded) well." |
|  |  |  | "*It’s partly because I know them well and for one reason or another they trust my recommendations.* I haven’t had any push back at all." |
|  |  |  | "My experience has been that they’re not particularly attached to one or the other. So if I say we’re going to switch it around for this reason, there’s not a lot of personal attachment." |
|  |  |  | "I have not had any patients question or want to know more when I explain that there’s some concern for increase in pneumonia." |
| **Pro 205** |  | **--** | "A lot of them are just like, ‘ok doc, I’ll give it a try, let’s see how it’s going’...so far patients haven’t come back to me saying, ‘no put me back on the steroids’." |
| **Pro 206** |  | "Because their prior doctors put them on this medicine, some of them are a little averse to change." | "I have been able to get several folks off of their steroid inhalers for the most part." |
|  |  | "Yeah, so some of my patients, their Pulmonologists happen to be community, and yes, they tend to be more liberal with the steroids. So that’s difficult, because they’re seeing the expert out there who wants them to be on it. So I have lost some battles with that one." | "I haven’t had anybody cling to it." |
|  |  |  | "*I just tell them, ‘the lung specialists recommend that we transition you off of this type of medicine because it potentially isn’t helping you as much as we thought it would’*. And they’re generally open to it... It’s not that they don’t want to come off of the steroids, it’s that they get confused." |

Key: Alternating color coded represent a different speaker to help visualize the range of quotes said by each speaker. Italics represent a potential strategy used to aid in deprescribing ICS. “--” Indicate no applicable quotes were found from the interview.

Table S2c: Matrix of quotes from patients regarding their reactions when their provider proposed discontinuing ICS

|  | **Reported Resistance** | **Reported Acceptance** |
| --- | --- | --- |
| **Pat A** | -- | "They told me to quit taking one of them, but I get confused over which they told me not to take, so I just don’t take either one of them…I think they only listed one of them, but I figured it was easier if I just don’t take any of them." |
|  |  | "Most of the time, I don’t even pay too much attention to it. I just do what the doctor says so they don’t yell at me." |
|  |  | "I go along with what the doctors tell me to do." |
|  |  | "Well, whatever the doctors think, because they have more schooling than I’ve got, and know more about stuff." |
| **Pat B** | "But it’s like a security blanket, just to have it here in case I should get some kind of a scenario." | "Actually, the other one was, it had a menthol flavor to it, and I preferred that to the one I’m using now, but I just got used to it." |
|  |  | "No, but it didn’t cause me any problems, so I didn’t debate it." |
|  |  | "Like I said, I’d just try it. Whatever happens, happens." |
| **Pat C** | -- | "My physician said something about long term use of Symbicort wasn’t recommended even though I’d been on it for a couple of years. They wanted me to change because of that. That’s all I know about it." |
| **Pat D** | -- | "The VA doctors decided that there was something in it that I shouldn’t be taking. I think there was a steroid in it, he was trying to get me off of steroids." |
| **Pat E** | -- | "I don’t remember exactly what was said, I don’t think there’s a whole lot said about it. She just wanted to make sure that, since it was recommended that I go to a different medication, that I would take it. I said that was fine. That’s pretty much it, I don’t recall anything else." |
|  |  | "Everything seems to be going good." |
| **Pat F** | -- | "They never told me why they were making the switch, they just said that they thought it’d be better to switch it." |
|  |  | "I’m used to the doctor saying that they think this will work better, and then I’ll try it for a while and then if it doesn’t work, that’s when I raise holy hell… So far it seems like it works alright?" |
| **Pat G** | -- | "He’s (my doctor is) really a straight shooter and I have a lot of respect for him, I guess, so I do everything that he says." |
| **Pat H** | -- | "As long as it makes me keep breathing and I’m doing fine, that’s fine. It doesn’t bother me that way, no." |
| **Pat I** | -- | -- |

Key: Alternating color coded represent a different speaker to help visualize the range of quotes said by each speaker. “--” Indicate no applicable quotes were found from the interview.
