# Supplementary material for: Provider anticipation and experience of patient reaction when deprescribing guideline discordant inhaled corticosteroids
Source: PLoS One. 2020 Sep 17;15(9):e0238511. doi: 10.1371/journal.pone.0238511 (PMC7498097; doi:10.1371/journal.pone.0238511)
Supplement: Attachment — Submitted filename: Author Response to Reviewers.docx [file pone.0238511.s007.docx]

**Response to Reviewers**


**Reviewer #1:** This is a very interesting article and the results contribute to the field. There are areas of the methods that require greater detail and I have a number of other comments which I think will improve the clarity and usefulness of the article to readers.

- Thank you for your feedback and the opportunity to respond to it.

Title – Suggesting mentioning ICS in the title to increase the descriptiveness – potentially also with the detail that it was guideline disconcordant use of ICS.

- Thank you for this feedback. We have changed the title to more accurately reflect the focus of our analysis. *“Provider anticipation and experience of patient reaction when deprescribing guideline discordant inhaled corticosteroids”*

Abstract – Suggest adding a summary of the results from the patient interviews (currently only mentions that they accepted the recommendation).

- We have followed your suggestion.

Introduction
The reference to Reeve’s focus group study are not completely accurate – the barriers are appropriately described, but the enablers found in the study are not mentioned. In the text this study is contrasted with another which reported openness to deprescribing which makes it seem like no willingness was reported in these focus groups which is not true.
Additionally, you may want to include reference to the studies using the Patients’ Attitudes Towards Deprecribing (PATD/rPATD) questionnaire regarding patient willingness to have a medication deprescribed. Including in some groups on specific drugs
e.g.
Edelman, M, et al. "Patients’ Attitudes Towards Deprescribing Alpha-Blockers and Their Willingness to Participate in a Discontinuation Trial." Drugs & aging 36.12 (2019): 1133-1139.
Reeve, E, et al. "Assessment of attitudes toward deprescribing in older Medicare beneficiaries in the United States." JAMA internal medicine 178.12 (2018): 1673-1680.

- Thank you for this suggestion. We have included both of these studies in the introduction (lines 112-122). However, we would like to emphasize that the Reeve et. al. asks for general views on medication discontinuation while the Edelman et al. article elicits patient response to a hypothetical question if the provider proposed discontinuation. However patient reaction (as reported by patients and anticipated/experienced by providers) has not been studied in the specific context of a deprescribing intervention.

Methods
It’s not clear why PCPs who had not been in the intervention were recruited for this study. Their perception of how a patient might react seems less important compared to PCPs who have recently conducted deprescribing of a ICS with a patient.

- Thank you for the opportunity to clarify this important point. The current analysis was done as a continuation of previously published work (Stryczek et. al.). We have referenced it in the manuscript several times (<https://link.springer.com/article/10.1007/s11606-019-05193-2>). This body of work includes providers unexposed to the intervention to understand their pre-existing attitudes and perspectives, as well as the perspectives of intervention exposed providers. The unexposed providers’ perspectives were equally informative for understanding patient reaction as a barrier to de-prescribing as were the intervention providers. The same interview guide from Stryczek et al. was used to assess unexposed providers views.

It appears that the findings reported here are a sub-result of the interviews with PCPs – (De-implementing Inhaled Corticosteroids to Improve Care and Safety in COPD Treatment: Primary Care Providers' Perspectives) – this needs to be made clear in this article. This has important implications for how the analysis was conducted – e.g. are these results/themes created simultaneously with the others and then just reported separately here?

- Yes, the current analysis was done as a continuation of previously published work (Stryczek et. al. as referenced in the above comment and added to the manuscript) (lines 157-159). However, provider responses regarding anticipation of patient reaction was an emergent, but preliminary finding identified in the unexposed providers. At the time of the first publication, there was not sufficient data to report these findings until data collection with intervention-exposed providers and patient interviews was completed for full analysis.

There are many recommended elements for reporting qualitative research missing in this article – for example, what was the relationship of the interviewer to the participants, how many people were involved in the coding, data saturation etc – see COREQ or other similar tools. Also, saturation is mentioned in the discussion but not in methods or results?

- We referred to the COREQ tool during the preparation of this manuscript and reported the applicable elements where appropriate.
- Team members responsible for qualitative data collection and coding were specified on the cover page and additional details were published in Stryczek et al.
- Regarding qualitative team member relationships with participants: some participants interviewed were clinician-researchers that have worked with members of the qualitative team (2 of 3 interviewers) in the past on other research projects. However, the interviewers are not clinicians, and interviews were completed with clinician-researchers within the scope of their clinician role. The interviewers were not working with participants on any projects at the time that the interviews were completed. No participants were involved in this project in any way.
- As far as recruitment for interviews, data collection for providers continued until no further participants volunteered, and data collection for patients continued until data saturation was reached. We have revised the methods to described this more clearly how saturation was determined.

Results
It is not clear which themes were deductively versus inductively created?

- The topic and themes presented in this manuscript were emergent findings. Meaning that we had noted preliminary findings in intervention unexposed providers anticipating patient reaction. We then looked at interviews from intervention exposed providers and patients to analyze if patient reaction was a prevalent topic in all three groups of interviews.

Patient resistance – while all the pts interviewed agreed to stop – authors report one person who had fear about stopping – it would be interesting to know if there was discussion of their decision making process, weighing up of pros and cons – why did the person who had fears not debate the decision?

- This was a very interesting point. The patients that we interviewed did not elaborate on their decision-making process, even with appropriately grounded prompts and probing. During the interviews, patients reported deferring to their providers.

“The post-intervention surveys confirmed that generally patients were receptive to the conversation regarding stopping an ICS or substituting with another drug.” – please add the detail that this was according to providers not patients.

- We clarified this as suggested.

Very little results from patients seem to be presented especially in comparison to the interview guide in the supplementary material – are the authors planning on publishing other aspects from the interviews elsewhere. E.g. experience of the conversation, decision making process?

- We state the goals of this particular analysis on Page 6 last paragraph (lines 151-159) starting with, “Our goal….” We included all the pertinent quotes relating to this analysis from provider and patient interviews. Despite the comprehensive interview guide, patient responses were brief and to the point without much elaboration. As stated above, patients did not elaborate on decision making process despite probing from interviewers. At this time, the authors are unclear if further data from the patient interviews will sufficiently add to future manuscripts.

Discussion
“Providers reported proposing discontinuing the medication on a trial basis, allowing the patient to return using the ICS if there are negative consequences, or anticipating broaching the topic not as a decision by the PCP but a conversation with the patient.” – this note of trial basis and restarting is not stated anywhere in the results?

- Thank you for this observation. We have amended the results section so that our discussion elaborates on the results. We added a specific quote where one provider mentions discontinuing an ICS on a trial basis. (lines 382-385)

“Further support in de-implementing interventions could target these instances to make the intervention more successful.” – Given that 46% of exposed providers never approached the topic what does that mean in the context of the intervention in the parent study – some discussion of what this intervention was would help put these results in context – i.e. a brief description of the DISCUSS COPD program. This is highlighted in the conclusion but not really mentioned in results or discussion.

- Thank you for pointing this out. Our intervention was a proactive e-consult generated by the pulmonary team targeted to primary care providers for recommendations to discontinue ICS when appropriate or other relevant recommendations. This is now explained in detail under Materials and Methods (lines 164-170). While preliminary data suggests that the e-consults were widely accepted, we cannot yet put the survey data into the context of how successful the e-consult intervention was. Our survey was not able to provide further context for this finding.

The authors note in the introduction that 2 previous studies have conducted qual study in response to a deprescribing intervention – I would have appreciated a specific discussion of how the results of this study compared with these others - 13. Martin P, Tannenbaum C. A realist evaluation of patients’ decisions to deprescribe in the 543 EMPOWER trial. BMJ Open. 2017 May;7(4):e015959. 14. Walsh K, Kwan D, Marr P, Papoushek C, Lyon WK. Deprescribing in a family health team: a study of chronic proton pump inhibitor use. J Prim Health Care. 2016;8(2):164.

- Thank you for this close observation. Upon review, the Walsh et. al. study reported findings from post-intervention provider surveys on the user feasibility and acceptability of the intervention rather than provider and patient experience as a result of the intervention. We have removed it as a reference. The Martin and Tannenbaum (2018) study has now been referenced in both the introduction and discussion (lines 448-451).

Limitations – Suggest adding that there were no NPs and no PAs in intervention exposed participant groups.

- Thank you for pointing this out. It is correct that we did not have PAs in our sample, but we did have NPs enrolled in our intervention. We were able to recruit two NPs for unexposed provider interviews but none for the 6 interviews we did with intervention-exposed providers. We believe that this was a product of small sample size, which we address in our limitations

Conclusion –
Recommend reducing the length and focusing on the actual conclusions that directly result from this study (the first paragraph of the conclusion repeats background and aims).

- Thank you for this suggestion. We have attempted to reduce the length and focus on the actual conclusions; we removed the first summarizing paragraph from the conclusion.

**Reviewer #2:** Thank you for the opportunity to review your manuscript, “Patient reaction when deprescribing a chronic medication: Findings from a quality improvement project.” This manuscript discusses a mixed methods analysis of a quality improvement project focused on discontinuation of inappropriate inhaled corticosteroids at two VA health care systems. This manuscript was well written and adds to the literature by providing patient and provider perspectives about a specific deprescribing intervention, as opposed to responding to hypothetical scenarios.

- Thank you for reviewing the paper and opportunity to revise it.

Here are a few minor suggestions to add clarity to the manuscript:

Participants and recruitment: Recommend clarify how you purposively sampled primary care providers and patients.

- Thank you for this recommendation. We have included a more detailed description in the methods (lines 184-191, 193-202).
- “One cohort of eligible providers were recruited for qualitative interviews prior to delivery of the DISCUSS COPD deprescribing intervention. These providers were not exposed to the intervention (unexposed). The second cohort included eligible providers exposed to the DISCUSS COPD deprescribing intervention (intervention-exposed). These providers were recruited for qualitative interviews after they received at least three DISCUSS COPD e-consults. Qualitative data collection continued until there were no new responses from PCPs to participate. Both groups of PCPs (unexposed and intervention-exposed) were recruited for quantitative surveys after the intervention was implemented.” (lines 184-191)
- “Patients of intervention-exposed PCPs were also purposively sampled for qualitative interviews if they were identified by the DISCUSS COPD Pulmonology team for the deprescribing intervention and the intervention was documented in the provider-patient encounter.” (lines 195-195)

Table 3
Were the questions about prescribing or discontinuing ICS specific to COPD?

- The survey questions about prescribing or discontinuing ICS were specific to mild-to-moderate COPD. We have now included specific wording from the survey for increased clarity and transparency (lines 244-255).

It is difficult to interpret the results in the “if yes” section. For example, were they thinking back for the last 6 months? How did they report data if the worked with multiple patients on potentially stopping ICS?

- The authors also had similar concerns. It has been noted in the limitations section: “The survey item asking respondents how patients responded if the respondent had proposed discontinuing an ICS did not orient the respondent to a specific patient (e.g., ‘for the last patient whose ICS you proposed discontinuing…’), which may have introduced variation in how respondents interpreted the item.” (lines 493-496)
