# Supplementary material for: Provider anticipation and experience of patient reaction when deprescribing guideline discordant inhaled corticosteroids
Source: PLoS One. 2020 Sep 17;15(9):e0238511. doi: 10.1371/journal.pone.0238511 (PMC7498097; doi:10.1371/journal.pone.0238511)
Supplement: Attachment — Submitted filename: Response to Reviewers.docx [file pone.0238511.s008.docx]

Submission Date: July 31, 2020

Joerg Heber, Ph.D.

Editor-in-Chief

*Public Library of Science ONE*

Dear Dr. Heber and Esteemed Reviewers,

We thank you for this opportunity to revise our manuscript for improved clarity and transparency. We have compiled our responses to your comments in the table below.

Please let me know if I can provide any additional information.

Sincerely,

Toral Parikh, M.D., M.P.H.

Post-Doctoral Fellow, VA Puget Sound Health Care system

Senior Fellow, Division of Gerontology and Geriatric Medicine, University of Washington

[parikht@uw.edu](mailto:parikht@uw.edu)

Office: 206-277-4771

|  | Section | Reviewer | Comment | Author Response |
| --- | --- | --- | --- | --- |
| 0 | General | Editor | Please ensure the work is reported to the COREQ checklist, and please make it clear in the paper as a standalone document.  As Reviewer 1 points out, this is important for future research in this area. | Thank you for the opportunity to address reviewer concerns and comments. We have included a COREQ checklist and done our best to make this manuscript a standalone paper. |
| 1 | General | Reviewer 1 | I appreciate the important work reported in this article and the responses from the authors. I just have two outstanding comments that while the authors responded to in the 'responses to reviewers' section, the changes made to the manuscript didn't fully provide the clarification necessary. | Thank you for allowing us to respond to your concerns and further clarifying our methods. |
| 2 | General | Reviewer 2 | Thank you for the opportunity to re-review the manuscript, “Provider anticipation and experience of patient reaction when deprescribing guideline discordant inhaled corticosteroids.” Thank you for adding additional information throughout the manuscript. It is easy to read and includes pertinent information. Below are two minor suggestions: | Thank you for the suggestions. |
| 3 | Title | Reviewer 2 | The title is more descriptive but is now quite long. Consider whether it would be possible to summarize it further (e.g., Provider and patient perceptions when deprescribing guideline discordant inhaled corticosteroids.”) | Thank you for this recommendation. Unfortunately, truncating the title implies that we are going to address all patient and provider perceptions regarding deprescribing of ICS. That has been done in our previous publication. In this paper, we did a deeper and focused analysis on patient reaction as it was anticipated and experienced. We want our title to reflect that limited focus to avoid misleading our readers as we had with our original title. |
| 4 | Methods | Reviewer 2 | Line 227: Please clarify if “perspectives on medical overuse” was explored and not “perspectives on medication overuse.” Recommend adding “and.” | We have made the following revisions: “changes in their inhaler prescription, and perspectives on medication overuse” (Lines 231-232) |
| 5 | Methods | Reviewer 1 | Lack of clarity of inductive vs deductive themes and how this work correlates to the previously published article by Stryczek et al.  The methods reported in line 229-237 mention inductive and deductive content analysis – but it is not clear whether the theme presented in this article was a result of inductive vs deductive. I think it would help to provide some timeline/context which was somewhat covered in the response document (but no changes were made to the manuscript). E.g. to say the unexposed/pre-intervention interviews were conducted with deductive and inductive approach. And one very interesting emergent (inductive) theme from these was this theme of patient reaction. Therefore in the exposed provider and patient interviews these theme was further explored – so this manuscript presents the results of all 3 interviews in relation to this theme. i.e. did the theme of patient reaction come from the previous lit and then the subthemes were all inductive, or was the theme and sub-themes all from previous lit. Or was the theme and subthemes all inductive from the first lot of interviews (unexposed) and then the 2 second lots of interviews coded to these themes and sub-themes with or without further refinement?  This article still needs to be standalone without the readers having to read the previous article. | Thank you for allowing us to clarify this important point. We have added the following:  “One inductive theme that emerged amongst barriers reported by unexposed providers was anticipation of patient reaction when discussing the discontinuing or substituting the ICS. As stated earlier, the interview guides for unexposed providers, intervention exposed providers, and patients were iteratively refined to allow further elaboration on patient reaction.” (Lines 240-243)  “This analysis builds on previously published work on unexposed providers [10]. Patient reaction, however, was an emergent theme for which data collection from intervention-exposed providers and patient interviews had continued well after the initial publication. The current findings further explore this emergent theme of patient reaction and includes analysis of data from intervention-exposed providers and patient interviews. Given the depth and subtle but important variations of this theme, we present a detailed analysis after synthesizing and incorporating both qualitative and quantitative data.” (Lines 276-281) |
| 6 | Methods | Reviewer 1 | **Items from the COREQ checklist not covered.**  The authors provided information in their responses which have not been added to the article. The authors say "We referred to the COREQ tool during the preparation of this manuscript and reported the applicable elements where appropriate." But key info such as whether there was duplicate coding, relationships etc is not in this article. Again, this article still needs to be standalone without the readers having to read the previous article. I strongly suggest adding the COREQ checklist with appropriate information as a supplementary file to this manuscript. While this may not seem essential to the presentation of the results, having this information clearly available is essential for future research in this field (such as conducting systematic reviews). | We have included a completed COREQ checklist and updated necessary information in the methods to make this a stand-alone manuscript as recommended. Thank you.  “Interviews lasted approximately 20-30 minutes, were audio-recorded and transcribed verbatim to facilitate analyses. Data collection was completed by authors CG, GGS, and KCS, and research assistant SW. Qualitative software (ATLAS.ti 7; Scientific Software Development GmbH, Berlin, Germany) was used for data management and coding. Coding and analyses were completed by team members (CG, CDH, GGS, KCS, and TJP) involved in data collection and members of the larger qualitative team trained in medicine and health services research.” (Lines 210-215) |
